# Exploring the risk of severe outcomes and the role of seasonal influenza vaccination in pregnant women hospitalized with confirmed influenza, Spain, 2010/11-2015/16

**DOI:** 10.1371/journal.pone.0200934

**Published:** 2018-08-08

**Authors:** Clara Mazagatos, Concepción Delgado-Sanz, Jesús Oliva, Alin Gherasim, Amparo Larrauri, the Spanish Influenza Surveillance System

**Affiliations:** 1 CIBER Epidemiología y Salud Pública (CIBERESP), Institute of Health Carlos III (ISCIII), Madrid, Spain; 2 National Centre of Epidemiology, Institute of Health Carlos III (ISCIII), Madrid, Spain; Public Health England, UNITED KINGDOM

## Abstract

Based on previous observations during pandemics and seasonal epidemics, pregnant women are considered at risk of developing severe influenza outcomes after influenza infection. With the aim of preventing severe influenza illness, the World Health Organization (WHO) includes pregnant women as a target group for seasonal influenza vaccination. However, influenza vaccine uptake during pregnancy remains low in many countries, including Spain. The objectives of this study were to increase the evidence of pregnancy as a risk factor for severe influenza illness and to study the potential role of seasonal influenza vaccination in the prevention of severe outcomes in infected pregnant women. Using information from the surveillance of Severe Hospitalized Confirmed Influenza Cases (SHCIC) in Spain, from seasons 2010/11 to 2015/16, we estimated that pregnant women in our study had a relative risk of hospitalization with severe influenza nearly 7.8 times higher than non-pregnant women of reproductive age. Only 5 out of 167 pregnant women with known vaccination status in our study had been vaccinated (3.6%). Such extremely low vaccination coverage only allowed obtaining crude estimates suggesting a protective effect of the vaccine against influenza complications (ICU admission or death). Our overall results support that pregnant women could benefit from seasonal influenza vaccination, in line with national and international recommendations.

## Introduction

Influenza epidemics have a major public health impact, causing an estimated three to five million cases of severe illness worldwide and approximately 250,000 to 500,000 deaths annually [1]. Severe influenza illness can lead to increased hospitalization and mortality rates during seasonal epidemics and pandemics, which result in a considerable healthcare and socio-economic burden [2]. Elderly people, children under 5 years of age and individuals with underlying medical conditions are most likely to suffer complications following influenza infection [3]. Pregnant women are also considered to be at risk and, consequently, the WHO recommends the inactivated influenza vaccine for all pregnant women regardless of the trimester of pregnancy [4]. In countries considering the initiation or expansion of seasonal influenza vaccination programmes, pregnant women are one of the main priority groups [3]. Annual vaccination every influenza season is recommended for all high-risk groups with the aim of preventing severe disease and deaths related with influenza [3].

The high risk of severe outcomes during pregnancy has been described for both pandemic and seasonal influenza [5–7]. In the 1918 and 1957 pandemics, abnormally high mortality rates were reported for pregnant women [8–10]. Regarding seasonal influenza before the 2009 pandemic, pregnant women had a higher risk of influenza-related hospitalizations compared to non-pregnant or postpartum women, particularly in the late stages of pregnancy [11, 12]. During the influenza A(H1N1)pdm09 pandemic, pregnancy was considered to be an independent risk factor for influenza severity and studies from different countries reported that pregnant women infected with the pandemic virus were more likely to experience hospitalizations [13–16], admission to intensive care units (ICU) [16–18], maternal mortality [19–23], and adverse neonatal outcomes [14, 17, 24–26]. During influenza seasons after the 2009 pandemic, pregnant women have had a higher risk than non-pregnant woman of being hospitalized due to respiratory illnesses [27, 28].

In a recent review and meta-analysis of 152 observational studies, most of them reporting cases from the 2009 pandemic period, the risk of hospitalization was found to be 2.4 times higher in pregnant women compared to non-pregnant women, and no associations were found between pregnancy and mortality or any other severe influenza outcomes [29]. However, no studies initiated follow-up before contact with the healthcare system and the results may reflect pregnancy conferring no greater risk than the risk associated with other conditions [29]. Also, several biases were identified in ecological studies from this review and all outcomes other than hospitalization were based on a very low quality of evidence [29].

Further research was recommended in a report of a WHO working group evaluating influenza burden and vaccine efficacy to increase the evidence on pregnancy as a risk factor for severe influenza outcomes [30]. It remains unclear whether the high hospitalization rate observed in infected pregnant women is due to severity of the influenza illness or for other reasons related with the pregnancy and the prevention of complications during delivery.

In many European Union (EU) member states, seasonal influenza vaccination is recommended for the elderly (age varies by country), persons aged over 6 months with a chronic medical condition, healthcare workers and pregnant women [31, 32]. In Spain, persons aged 65 years or older have the highest vaccination rates, although a declining trend has been observed in recent years, mainly since the 2009 pandemic [33]. Influenza vaccine coverage in this group has ranged from 70.1% in season 2005/06 to 56.1% in season 2015/16. National Spanish data on influenza vaccine coverage for this risk group are available online [33, 34]. Other eligible groups for influenza vaccination in Spain have low coverage, such as individuals with chronic diseases [35] and healthcare workers [36]. In the limited studies reporting coverage on pregnant women in Spain, vaccination rates are extremely low, with pregnant women not even reaching 5% coverage [37, 38].

Despite uncertain data on the prevention of adverse birth outcomes [39], there is strong and consistent evidence that vaccination during pregnancy protects women and their newborns against influenza infection [40, 41]. The existing knowledge on the benefits of influenza vaccination during pregnancy as well as the safety of the vaccine, with no indication of harm or side effects in vaccinated women or their infants [40–43], strongly supports the routine administration of the seasonal influenza vaccine to all healthy pregnant women. However, providing new evidence of the risk of severe disease in pregnant women and the protective role of the vaccine during pregnancy could improve the adherence to annual vaccination every influenza season in this high-risk population in line with current official recommendations.

The surveillance system for Severe Hospitalized Confirmed Influenza Cases (SHCIC) was established in Spain after the 2009 influenza pandemic, and includes sentinel hospitals from all Spanish regions covering between 45% and 60% of the total Spanish population [44], which is around 46 million people [45].The system is based on the notification of laboratory-confirmed influenza cases that require hospitalization based on clinical severity and meet the SHCIC case definition (presenting with pneumonia, septic shock, acute respiratory distress syndrome (ARDS), multiple organ dysfunction syndrome (MODS) or admission to ICU). In this study, we have used the information obtained from the surveillance of severe hospitalized influenza cases in Spain during the post-pandemic period 2010–2016. Our main objectives were to increase the evidence of pregnancy as a risk factor for severe influenza illness and to study the potential role of seasonal vaccination preventing severe influenza illness in pregnant women. Raising awareness on the risk of seasonal influenza during pregnancy and on the potential protective effect of the influenza vaccine against severe influenza is critical to support current national and international recommendations regarding influenza vaccination of pregnant women.

## Materials and methods

### Surveillance of Severe Hospitalized Confirmed Influenza Cases (SHCIC)

Data were obtained from the surveillance of Severe Hospitalized Confirmed Influenza Cases (SHCIC) in Spain during influenza seasons 2010/11 to 2015/16, including hospitals from all Spanish regions covering approximately 21 to 28 million people, nearly half of the Spanish population. Although the SHCIC surveillance began during the 2009 pandemic, the system was fully established in the following seasons. Data from the pandemic period were not directly comparable and excluded from this study. Clinicians at the participating hospitals were recommended to swab any patient who presented with influenza-like illness (ILI) and required hospitalization. The system is based on the notification of hospitalized laboratory-confirmed influenza cases in all wards who met the Spanish SHCIC case definition, as follows: “Any case with clinical features compatible with influenza, requiring hospitalization for clinical severity and presenting with at least one of the following criteria: pneumonia, septic shock, ARDS, MODS, or admission to ICU”.

Clinical, epidemiological and virological data were collected, along with information on the patient outcome: age, sex, dates of hospitalization and symptom onset, virus type and subtype, presence of underlying medical conditions [(class III obesity (BMI≥40 kg/m^2^), chronic respiratory, cardiovascular, renal and liver diseases, diabetes mellitus, and immunosuppression)], complications (pneumonia, secondary bacterial co-infection, ARDS and MODS), antiviral treatment (yes/no), delay in the start of treatment from symptom onset (48 hours or less vs. more than 48 hours), influenza vaccination status (yes/no, date of vaccination), admission to ICU (yes/no), death during hospital stay (yes/no), Spanish region and influenza season.

### Characterization of the study population

From all patients reported as SHCIC, we selected women of reproductive age, aged between 15 and 49 years, as our study population. The proportion of patients with each variable was calculated excluding records with missing or unknown data for that variable. We compared demographic, clinical and virological characteristics of women of reproductive age, pregnant and non-pregnant, who were hospitalized with severe confirmed influenza with median test, the χ^2^ test or Fisher’s exact test, as appropriate. We classified our population of women in two age groups, 15–34 and 35–49 years.

### Risk of severe influenza outcomes in women of reproductive age

We used multivariable logistic regression and calculated the odds ratio (OR) and the corresponding 95% confidence interval (CI) in women of reproductive age who were identified as SHCIC, identifying factors associated with the risk of: (1) ICU admission; (2) ICU admission or death; and (3) death only. When analyzing pregnancy as a risk factor, we performed an additional sensitivity analysis in which we considered 74 women who had missing or unknown information regarding pregnancy status and included them in the non-pregnant group. Both analyses were adjusted for the following confounding factors: age group (15–34 and 35–49 years), virus type/subtype, class III obesity, other underlying medical conditions (one or more), and delay in antiviral treatment (>48 hours from symptoms onset).

### Severe hospitalized pregnant women with confirmed influenza

**Fig 1:**
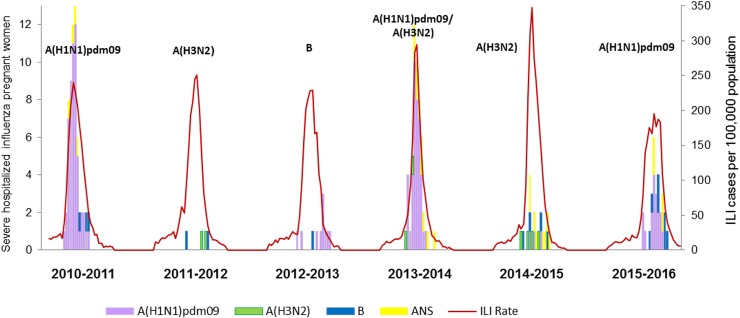
Weekly ILI rates and number of severe hospitalized pregnant women with confirmed influenza by type/subtype of influenza virus, Spain, seasons 2010/11 to 2015/2016.

The weekly number of pregnant women among the SHCICs during the study period was plotted together with the weekly ILI rates, obtained from the Spanish primary care Influenza Sentinel Surveillance System (SISSS). (Fig 1.) [46]. Week in refers to week of hospitalization.

SHCICs in pregnant women were further classified by the level of severity in two groups: first, pregnant women with less serious outcomes, who did not require ICU admission and survived; and second, those with the most severe outcomes, who were admitted to ICU and/or died. Demographic, clinical and virological characteristics of both groups were compared to detect any significant differences using OR and 95% CI.

### Effect of the vaccine in pregnant women

Multivariable logistic regression models were used to estimate the effect of seasonal vaccination on influenza outcomes and complications in severe hospitalized pregnant women with confirmed influenza. We adjusted for the following potential confounding factors: age, virus type/subtype, class III obesity, other underlying medical conditions (one or more), and delay in the start of antiviral treatment (>48 hours from symptoms onset).

### Relative risk of hospitalization in pregnant women

We calculated the relative risk of hospitalization in pregnant women comparing the rates of hospitalization in women of reproductive age with severe confirmed influenza that were pregnant with those who were not pregnant. To calculate the rates of hospitalization in pregnant women we used as the numerator the number of pregnant SHCIC women and as the denominator an estimation of the annual prevalence of pregnancy in the hospital catchment population obtained by extrapolation of this prevalence in general population. The number of pregnant women in the general population was estimated following previously published methods [15, 16, 47], with national fertility rates from the starting year of each season, 2010 to 2015, and adjusting by abortions reported in Spain for these years (information from 2016 was not available). A pregnancy resulting in a live birth lasts 9 months, while a pregnancy resulting in abortion lasts 2 months on average. For each year, we added 9/12 of the fertility rate to 2/12 of the abortion rate, and multiplied the result by the population of women of reproductive age, getting an estimation of the number of pregnant women in the Spanish general population. To calculate the hospitalization rates of influenza confirmed non-pregnant women, we used as the numerator the number of non-pregnant SHCIC women, and as the denominator the number of non-pregnant women in the catchment hospital population, obtained by subtracting the previously estimated number of pregnant from the total population of women of reproductive age. Thus, the relative risk of hospitalization in pregnant women pregnancy was calculated as follows:

$$RR=\frac{\frac{Pregnant\;women\;hospitalized\;as\;SHCIC}{Pregnant\;women\;aged\;15\;to\;49\;in\;the\;hospital\;catchment\;population}}{\frac{Non\;pregnant\;women\;hospitalized\;as\;SHCIC}{Non\;pregnant\;women\;aged\;15\;to\;49\;in\;the\;hospital\;catchment\;population}}$$

Confidence intervals for the relative risks were calculated using MedCalc statistical software available online [48]. Population estimates and annual fertility rates (live births per 1000 women aged 15–49 years) were obtained from the Spanish National Statistics Institute [49], and abortion rates (voluntary terminations of pregnancy per 1000 women aged 15–44 years) were provided by the Ministry of Health, Social Services and Equality [50].

### Statistical analysis

For all statistical analysis, 2-sided p values of ≤0.05 were considered statistically significant. All statistical analyses were performed using the Stata 14.0 software (Stata Corp., College Station, U.S.).

### Ethical approval

This study was conducted in the frame of the existing Surveillance of SHCIC at the National Centre of Epidemiology. Formal ethical approval is not required for routine surveillance activities in Spain. However, anonymized data were collected and verbal consent was obtained from all the patients before swabbing for surveillance purposes.

## Results

### Women of reproductive age hospitalized with severe influenza

#### Characteristics of pregnant vs. non-pregnant women

During the six-season period, 1,060 women aged between 15 and 49 years were reported as SHCIC. Among those 176 were pregnant, 810 were not pregnant and 74 had unknown pregnancy status. The majority of pregnant women were in the 15–34 year age group (69.3%) with a median age of 31 years vs. 40 years for non-pregnant women (Table 1). Influenza A(H1N1)pdm09 virus was responsible for 83.3% and 77.5% of infections in pregnant and non-pregnant women, respectively. Influenza A(H3N2) infection was less frequent in those pregnant (5.3% vs. 12.1%) while influenza B was similarly observed in the pregnant and non-pregnant groups (11.3% vs. 10.4%). These differences in the distribution by type and subtype of influenza virus were not statistically significant (p = 0.06) (Table 1).

**Table 1:** Demographic and clinical characteristics of hospitalized confirmed severe influenza women of reproductive age, by pregnancy status. Spain, 2010–2016.

	Pregnant	Not pregnant	
	N = 176	N = 810	
Characteristics	No. (%)	No. (%)	p value
Median age (years); IQR	31; 29–35	40; 33–45	<0.001
Age group (years)			<0.001
15–34	122 (69.3)	229 (28.3)	
35–49	54 (30.7)	581 (71.7)	
Virus type and subtype			0.06
A(H1N1)pdm09	125 (83.3)	482 (77.5)	
A(H3N2)	8 (5.3)	75 (12.1)	
B	17 (11.3)	65 (10.4)	
Underlying medical conditions *			
Chronic respiratory disease	8 (5.6)	86 (15.6)	0.002
Chronic renal disease	0 (0.0)	27 (3.9)	0.005
Chronic liver disease	0 (0.0)	31 (4.5)	0.002
Chronic cardiovascular disease	4 (2.4)	36 (5.3)	0.15
Immunosuppression	1 (0.6)	121 (17.6)	<0.001
Class III obesity (BMI ≥ 40kg/m^2^)	7 (4.2)	114 (16.9)	<0.001
Diabetes	4 (2.4)	63 (9.2)	0.002
Any of the above	23 (16.7)	373 (65.1)	<0.001
Complications *			
Pneumonia	119 (67.6)	647 (80.7)	<0.001
Co-infection	21 (15.1)	160 (27.0)	0.003
ARDS	39 (23.2)	211 (27.7)	0.23
MODS	7 (4.2)	63 (8.4)	0.07
Outcome			
ICU admission	64 (38.3)	320 (42.1)	0.37
Death	7 (4.4)	63 (8.2)	0.095
Seasonal trivalent influenza vaccine	5 (3.0)	40 (6.2)	0.10
Antiviral treatment	144 (84.7)	698 (88.6)	0.16
Antiviral started > 48 h. from symptoms onset	100 (75.2)	505 (78.8)	0.36
Hospitalization > 48 h. from symptoms onset	97 (61.0)	530 (71.3)	0.01

IQR: interquartile range; BMI: body mass index; ICU: intensive care unit.

* One or more.

Pregnant women of reproductive age were significantly less likely to have underlying medical conditions than non-pregnant women (16.7% vs. 65.1%, p<0.001). Specifically, pregnant women were less likely to have chronic respiratory disease (5.6% vs. 15.6%, p = 0.002), immunosuppression (0.6% vs. 17.6%, p<0.001), class III obesity (4.2% vs. 16.9%, p<0.001) and diabetes (2.4% vs. 9.2%, p = 0.002) than non-pregnant women. Also, they developed fewer complications such as pneumonia (67.6% vs. 80.7%, p<0.001) or co-infection (15.1% vs. 27.0%, p = 0.003). Although not significant, the proportion of outcomes resulting in admission to ICU and death was lower in the pregnant group (38.3% vs. 42.1% and 4.4% vs. 8.2%, respectively) (Table 1).

The seasonal inactivated influenza vaccine coverage was very low in all women of reproductive age, but particularly in the pregnant group, with only five women reported as vaccinated (3%). Pregnant women were significantly more likely to be hospitalized within 48 hours from symptom onset (61.0% pregnant women had delayed hospitalization vs. 71.3% non-pregnant, p = 0.01). More than 75% of hospitalized women, both pregnant and non-pregnant, received antiviral treatment more than 48 hours after symptoms onset (Table 1).

#### Factors associated with severe influenza outcomes

Our results obtained from the multivariable analysis showed that factors, independently associated with severe influenza in women of reproductive age were the following: a) Having one or more underlying medical condition is associated with a higher risk of ICU admission (OR 1.84, 95% CI 1.16–2.90), ICU admission or death (OR 1.85, 95% CI 1.16–2.95) and death (OR 2.76, 95% CI 1.12–6.84); b) Class III obesity (BMI≥40 kg/m^2^) is associated with a higher risk of ICU admission or death (OR 2.43, 95% CI 1.29–4.58); c) Delayed antiviral treatment started > 48 hours after symptoms onset is associated with a higher risk of ICU admission or death (OR 1.76, 95% CI 1.05–2.96) (Table 2). Also, women of reproductive age infected with either influenza A(H3N2) or B are less likely to be admitted to ICU or to die than those infected with influenza A(H1N1)pdm09 (OR 0.38, 95% CI 0.17–0.85 and OR 0.40, 95% CI 0.19–0.84, respectively) (Table 2).

**Table 2:** Factors associated with ICU admission, death and ICU admission or death in hospitalized confirmed severe influenza women of reproductive age. Spain, 2010–2016.

		ICU admission	ICU/ death	Death
**Age group**				
15–34 years old (ref.)		125 (35.7)	128 (38.0)	23 (6.6)
35–49 years old		292 (45.1)	300 (47.6)	53 (8.3)
	Crude OR (95%CI)	1.48 (1.13–1.93)	1.48 (1.13–1.94)	1.29 (0.77–2.14)
	Adjusted OR (95%CI) †	1.23 (0.76–1.97)	1.27 (0.79–2.06)	0.91 (0.38–2.21)
**Virus type/subtype**				
A(H1N1)pdm09 (ref.)		283 (45.8)	290 (48.2)	56 (9.3)
A(H3N2)		29 (38.7)	29 (39.7)	3 (3.7)
	Crude OR (95%CI)	0.75 (0.46–1.22)	0.71 (0.43–1.16)	0.37 (0.11–1.22)
	Adjusted OR (95%CI) †	0.43 (0.19–0.94)	0.38 (0.17–0.85)	0.71 (0.20–2.54)
B		27 (30.3)	29 (34.9)	4 (4.6)
	Crude OR (95%CI)	0.52 (0.32–0.83)	0.58 (0.36–0.93)	0.46 (0.16–1.31)
	Adjusted OR (95%CI) †	0.45 (0.22–0.92)	0.40 (0.19–0.84)	-
**Underlying medical conditions** *				
None (ref.)		146 (37.9)	146 (39.5)	15 (4.1)
One or more		159 (45.5)	165 (49.3)	38 (11.4)
	Crude OR (95%CI)	1.42 (1.06–1.91)	1.49 (1.10–2.01)	3.00 (1.62–5.55)
	Adjusted OR (95%CI) †	1.84 (1.16–2.90)	1.85 (1.16–2.95)	2.76 (1.12–6.84)
**Class III obesity (BMI ≥ 40kg/m**^**2**^**)**				
No (ref.)		268 (37.7)	274 (39.6)	45 (6.3)
Yes		80 (65.0)	80 (66.7)	12 (10.4)
	Crude OR (95%CI)	3.08 (2.06–4.59)	3.05 (2.03–4.59)	1.75 (0.89–3.41)
	Adjusted OR (95%CI) †	2.33 (1.26–4.30)	2.43 (1.29–4.58)	1.31 (0.45–3.82)
**Delay in antiviral treatment**				
< 48 hrs. from symptom onset (ref.)		66 (39.3)	68 (41.0)	17 (9.9)
> 48 hrs. from symptoms onset		266 (45.4)	272 (47.5)	43 (7.3)
	Crude OR (95%CI)	1.28 (0.91–1.82)	1.30 (0.92–1.85)	0.72 (0.40–1.30)
	Adjusted OR (95%CI) †	1.65 (0.99–2.75)	1.76 (1.05–2.96)	1.02 (0.41–2.52)
**Pregnancy**				
Non-pregnant (ref.)		320 (42.1)	331 (44.4)	63 (8.2)
Pregnant		64 (38.3)	64 (40.3)	7 (4.4)
	Crude OR (95%CI)	0.85 (0.61–1.20)	0.84 (0.59–1.19)	0.51 (0.23–1.14)
	Adjusted OR (95%CI) †	0.93 (0.51–1.66)	0.94 (0.52–1.70)	1.17 (0.36–3.78)
**Sensitivity analysis** (N = 1060) ‡				
Non-pregnant (ref.)		353 (42.5)	364 (45.1)	69 (8.4)
Pregnant		64 (38.3)	64 (40.3)	7 (4.4)
	Crude OR (95%CI)	0.84 (0.60–1.18)	0.82 (0.58–1.16)	0.50 (0.23–1.11)
	Adjusted OR (95%CI) †	0.91 (0.51–1.61)	0.92 (0.51–1.66)	1.21 (0.38–3.90)

OR: Odds ratio; CI: confidence interval; ICU: Intensive Care Unit.

* Underlying medical conditions excluding obesity: chronic respiratory disease, chronic renal disease, chronic cardiovascular disease, chronic liver disease, diabetes, and immunosuppression

† Adjusted for: age group (15–34 and 35–49 years), virus type/subtype, class III obesity (BMI ≥ 40kg/m^2^), other underlying medical conditions (one or more), and delay in antiviral treatment (>48 h. from symptoms onset).

‡ Considering missing data for pregnancy as “Not pregnant”. (“Pregnant” n = 176; “Not-pregnant” n = 884).

We did not find a statistically significant association between pregnancy and severe outcomes in terms of a higher risk of ICU admission or death (ICU: 0.93, 95% CI 0.51–1.66; ICU or death: 0.94, 95% CI 0.52–1.70; Death: OR 1.17, 95% CI 0.36–3.78). The results were similar in the sensitivity analysis, in which the risk of death slightly increased but it was still not significant (OR 1.21, 95% CI 0.38–3.90) (Table 2).

### Pregnant women hospitalized with severe influenza characteristics of pregnant women reported as SHCIC

The number of hospitalized pregnant women with severe confirmed influenza varied by season and type/subtype of the influenza virus (Table 3, Fig 1). During the six studied seasons, 176 pregnant women were hospitalized with laboratory-confirmed severe influenza. Most of these cases were hospitalized during seasons 2010/11 (35%), 2013/14 (30%) and 2015/16 (16%), when influenza virus A(H1N1)pdm09 was either predominant or co-circulating. (Table 3, Fig 1).

**Table 3:** Severe outcomes in pregnant women hospitalized with severe confirmed influenza infection, by season. Spain, 2010–2016.

Season	Predominant influenza virus	Pregnant	Non-ICU / Non-death	ICU admission alive	Death
		No. (%)	No. (%)†	No. (%)†	No. (%)†
2010–2011	A(H1N1)pdm09	61 (35)	29 (57)	19 (37)	3 (6)
2011–2012	A(H3N2)	4 (2)	3 (75)	1 (25)	0 (0)
2012–2013	B	11 (6)	5 (45)	6 (55)	0 (0)
2013–2014	A(H1N1)pdm09 / A(H3N2)	52 (30)	35 (70)	12 (24)	3 (6)
2014–2015	A(H3N2)	19 (11)	7 (50)	7 (50)	0 (0)
2015–2016	A(H1N1)pdm09	29 (16)	16 (55)	12 (41)	1 (3)
Total No. (%)		176 (100)	95 (59.8)	57 (36)	7 (4)

ICU: Intensive Care Unit

† 17pregnant had incomplete information for severe outcomes (N = 159).

15 (9%) pregnant women hospitalized with severe confirmed influenza were in the first trimester of pregnancy, 50 (31%) were in the second and 94 (59%) were in the third. The remaining 17 pregnant women had missing information about week of gestation. For all hospitalized pregnant women reported during the six seasons, 57 (36%) were admitted to ICU and survived, and 7 (4%) died, all of them previously admitted to ICU (Table 3).

The characteristics of influenza infections in severe hospitalized pregnant women are presented by severity level in Table 4. Pregnant women with the most severe outcomes (who were admitted to ICU and/or died), were slightly older than those who did not require ICU admission and survived (median age 33 vs. 31), and they were more likely to have underlying cardiovascular disease. They presented with more pneumonia and ARDS than pregnant women with less serious outcomes (Table 4). Only one (1.7%) of the pregnant women with the most severe outcomes and three (3.3%) with less serious outcomes had received the seasonal trivalent influenza vaccine. A higher proportion of pregnant women with the most severe outcomes were treated with antivirals (93.6% vs. 78.3%, p = 0.01), and they were hospitalized and received the antiviral treatment later than pregnant women with less serious outcomes (71.7% vs. 54.2%, p = 0.03 and 86.0% vs. 67.1%, p = 0.02, respectively) (Table 4).

**Table 4:** Demographic and clinical characteristics of pregnant women hospitalized with severe confirmed influenza infection, by level of severity. Spain, 2010–2016.

All pregnant	No ICU admission and no death	ICU admission or death	
N = 176	N = 95	N = 64	
Characteristics	No. (%)	No. (%)	p value
Median age (years); IQR	31; 28–35	33; 30–37	<0.001
Age group (years)			0.10
15–34	71 (74.7)	40 (62.5)	
35–49	24 (25.3)	24 (37.5)	
Virus type and subtype			0.23
A(H1N1)pdm09	64 (79.0)	48 (88.9)	
A(H3N2)	5 (6.2)	3 (5.6)	
B	12 (14.8)	3 (5.6)	
Underlying medical conditions *			
Chronic respiratory disease	5 (5.9)	3 (6.4)	0.91
Chronic renal disease	0 (0.0)	0 (0.0)	-
Chronic liver disease	0 (0.0)	0 (0.0)	-
Chronic cardiovascular disease	0 (0.0)	3 (5.0)	0.03
Immunosuppression	1 (1.1)	0 (0.0)	0.42
Class III obesity (BMI ≥ 40kg/m^2^)	2 (2.2)	5 (8.3)	0.08
Diabetes	2 (2.2)	1 (1.7)	0.85
Any of the above	11 (13.4)	10 (22.7)	0.18
Complications *			
Pneumonia	56 (59.0)	56 (87.5)	<0.001
Co-infection	10 (12.2)	9 (20.0)	0.24
ARDS	2 (2.2)	35 (56.5)	<0.001
MODS	0 (0.0)	7 (11.9)	0.001
Seasonal trivalent influenza vaccine	3 (3.3)	1 (1.7)	0.56
Antiviral treatment	72 (78.3)	58 (93.6)	0.01
Antiviral started > 48 h. from symptoms onset	47 (67.1)	43 (86.0)	0.02
Hospitalization > 48 h. from symptom s onset	45 (54.2)	43 (71.7)	0.03

Among 176 pregnant women, 17 had incomplete data for severe outcomes (N = 159)

IQR: interquartile range; BMI: body mass index; ICU: intensive care unit.

* One or more.

#### Effect of the vaccine against severe influenza in pregnant women

Our results revealed very low adherence to vaccination in the pregnant group. Only five pregnant women were vaccinated and of those, one was admitted to ICU, three had pneumonia and none of them died (Table 5). The small sample size and extremely low vaccination coverage did not allow for an adjusted analysis of the vaccine effectiveness against severe outcomes or complications.

**Table 5:** Effect of the seasonal influenza vaccination on influenza outcomes and complications in pregnant women hospitalized with severe confirmed influenza infection. Spain, 2010–2016.

	Unvaccinated No. (%)	Vaccinated No. (%)	Crude OR (95% CI)
Pregnant women (N = 176)*	162	5	
ICU admission	58 (98.3)	1 (1.7)	0.41 (0.05–3.79)
Death	7 (100)	0 (0.0)	-
ICU admission or death	58 (98.3)	1 (1.7)	0.51 (0.05–5.04)
Pneumonia	108 (97.3)	3 (2.7)	0.75 (0.12–4.62)

OR: Odds ratio; CI: confidence interval; ICU: Intensive Care Unit.

*9 pregnant women had unknown vaccination status and were not included in this table.

### Relative risk of hospitalization with severe influenza in pregnant women

**Fig 2:**
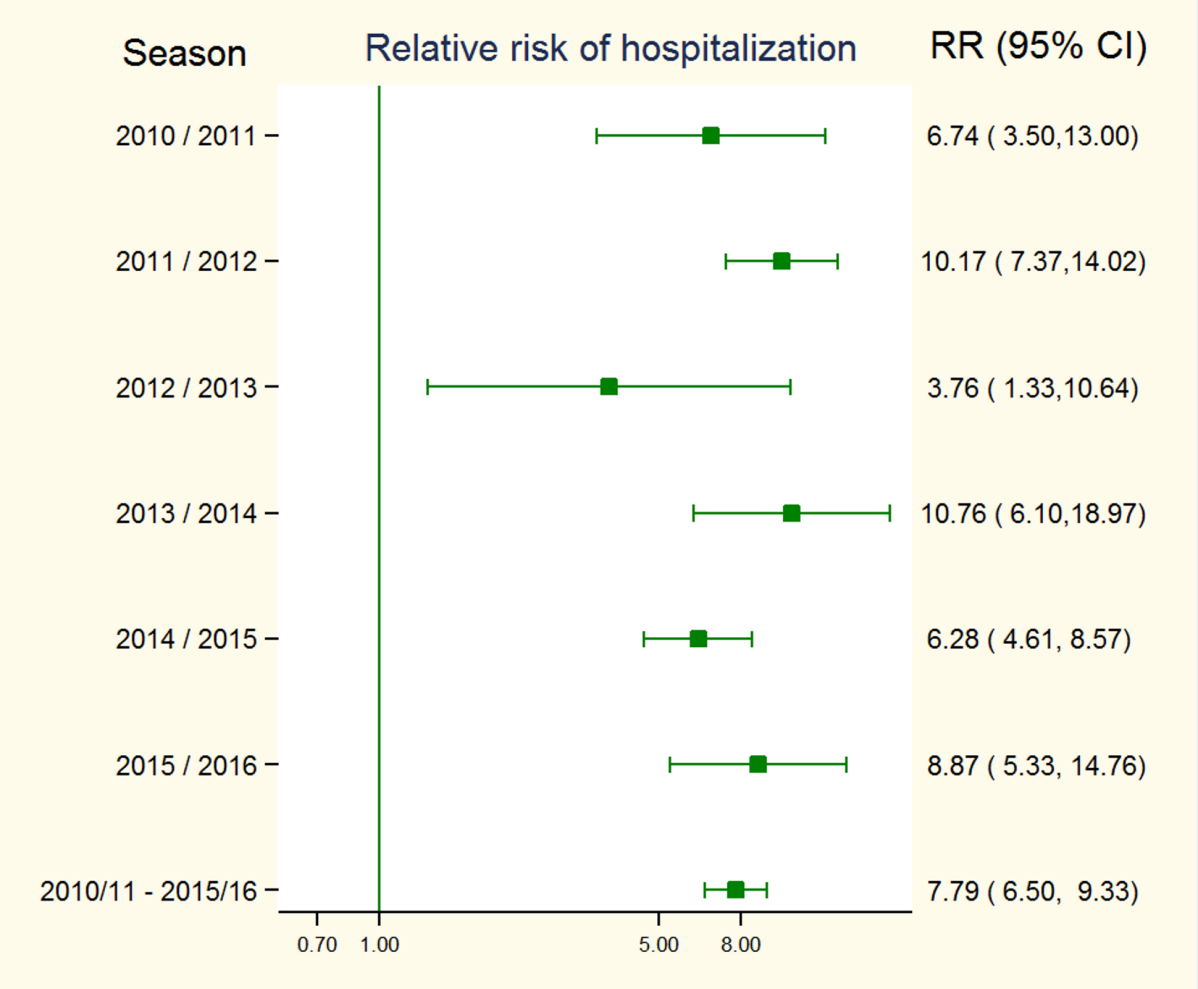
Relative risk of hospitalization with severe influenza in pregnant women, Spain, 2010–2015.

The risks of hospitalization varied by year, but pregnant women always had a higher risk than non-pregnant women (Table 6). The highest relative risks of hospitalization for pregnant women were estimated during seasons 2011/2012, 2013/2014 and 2015/2016. For the whole period (2010–2015), excluding data from 2016, we estimated that the relative risk of hospitalization with severe influenza for pregnant women was 7.8 (CI: 6.5–9.3) (Fig 2).

**Table 6:** Risk of hospitalization with laboratory-confirmed severe influenza infection in women of reproductive age, according to pregnancy status. Spain, 2010–2016.

	Estimated pregnancy rate (per 1000 women)	Women of reproductive age (15–49 years) in the hospital catchment population			SHCIC in women of reproductive age		Risk of hospitalization with severe influenza	
Season*		Women in hospital catchment population	Pregnant women	Non-pregnant women	Pregnant women	Non-pregnant women	In pregnant women	In non-pregnant women
2010/11	33.6	6,590,991	221,175	6,369,816	11	47	5.0	0.7
2011/12	33.0	4,909,608	162,118	4,747,490	50	144	30.8	3.0
2012/13	32.2	4,493,163	144,480	4,348,683	4	32	2.8	0.7
2013/14	30.7	4,982,494	152,852	4,829,642	16	47	10.5	1.0
2014/15	31.1	4,810,222	149,593	4,660,629	48	238	32.1	5.1
2015/16	31.0	4,946,710	153,262	4,793,448	19	67	12.4	1.4
Total	31.9	30,733,188	983,480	29,749,708	148	575	15.0	1.9

SHCIC: Severe hospitalized laboratory-confirmed influenza cases

* For all calculations we used data from the first year of each season

## Discussion

We have estimated that the risk of severe influenza-related hospitalization is approximately eight times higher in pregnant women than in non-pregnant women of childbearing age in Spain. Using the information from the Spanish influenza surveillance system at hospital level, we found however no association between pregnancy and the risk of ICU admission or death in women hospitalized with confirmed influenza infection. Even though the number of vaccinated women in our study is too small to make definitive conclusions about the vaccine effectiveness against severe influenza during pregnancy, maternal influenza immunization has a positive effect both in mothers and infants [40, 41, 51], and our results suggest that pregnant women in Spain could benefit from this public health intervention.

In the years following the 2009 pandemic, different countries reported high hospitalization rates for pregnant women infected with the influenza A(H1N1)pdm09 virus. [15, 16, 24, 52, 53]. Hospitalizations in pregnant women were also considerable during seasonal pre-pandemic epidemics, with women in the third trimester of pregnancy being at the highest risk [11, 12]. Therefore, our results are in line with previous observations and confirm the impact of influenza on hospitalizations of pregnant women in Spain during an inter-pandemic period.

Even though infected pregnant women are clearly at higher risk of being hospitalized with severe disease, we did not find an association between seasonal influenza during pregnancy and the risk of ICU admission or death in the studied period. These results are similar to those reported in a study of pregnant women hospitalized during the 2009 pandemic in the UK [54], where pregnancy increased the likelihood of hospital admission, but not severe outcomes. Another study from Spain, not using our SHCIC case definition, compared infected non-vaccinated pregnant and non-pregnant women who were hospitalized during the 2009 pandemic, and also found no significant differences in symptoms or the risk of influenza complications among both groups of women [55]. Nevertheless, in contrast with these observations, many other studies have associated influenza infection in pregnancy with an increased risk of severe outcomes, mostly during pandemics [6]. After the 2009 pandemic, high mortality rates in pregnant women were reported in many countries, like the USA [15, 21], the UK [22], Turkey [20] and South Africa [56]. Also, pregnant women had a higher risk of being admitted to ICU in Australia [16, 17] and France [18]. A possible explanation for these conflicting results could be the different prevalence of underlying comorbidities between studies and the different strategies for the control of this important confounding factor. Also, these articles suggesting a higher risk of severe illness during pregnancy referred mostly to the 2009 influenza pandemic, when health systems were considerably stressed, and they often describe limitations related to a biased reporting of severe cases.

The existing evidence supporting pregnancy as a risk factor for severe outcomes such as ICU admissions or deaths is mostly based on individual observational studies reporting cases from the 2009 pandemic. When reviewing the data in large systematic reviews, and including information from seasonal influenza epidemics, the evidence associating pregnancy with severe illness in mothers and infants seems to be more limited [29, 30].

It is not fully clear whether the increased influenza-related hospitalization rates in pregnant women during inter-pandemic seasons are related to the severity of the disease or to other pregnancy-related factors. What our results would suggest, in line with others [29, 30], is that pregnancy may not be independently associated with severe outcomes but rather with a higher risk of precautionary hospital admission, with pregnant women being more likely to be hospitalized than other women with similar degree of illness, especially during late stages of pregnancy. However, pregnant women in our study had to meet clinical severity criteria for their inclusion in the Spanish surveillance system at hospital level. Even if they had significantly less comorbidities or complications than non-pregnant women, they were all severe cases, and this supports the idea that some pregnancy-related factors might be responsible for the hospitalization with severe influenza infection.

Physiological changes leading to an increased risk of severe influenza during pregnancy are still present in women up to 2 weeks postpartum, and treatment with antivirals is recommended for them also, together with pregnant women, if they have suspected or confirmed influenza [57]. The SHCIC surveillance only collects information about current pregnancies and in this study we might be including post-partum women in the non-pregnant group, in contrast with other studies in which they are analyzed as a risk group similar to pregnant women [17, 21].

One of the factors we found to be associated with a higher risk of ICU admission in women of reproductive age was the delay in the start of the antiviral treatment. Also, when comparing the outcomes in pregnant women, those with the most serious outcomes (ICU and/or death) were hospitalized and received antiviral treatment later than those who did not require ICU and survived. These results suggest the importance of early administration of antiviral treatment to prevent severe influenza outcomes.

Despite recommendations, influenza vaccination during pregnancy remains low in Spain. Although there is no official data on influenza vaccination in pregnant women, two studies have reported coverage lower than 5% for different Spanish regions [37, 38]. In our study, only 3.6% of all pregnant women hospitalized with severe confirmed influenza had received the influenza seasonal vaccine. This poor compliance with official recommendations in Spain may be due to a combination of factors, including perception that their risk of seasonal influenza infection is low, considering the vaccine unnecessary, misconceptions about vaccine efficacy and safety during pregnancy, and unawareness of the current influenza immunization recommendations among obstetricians, especially in relation to the first trimester of pregnancy [37, 58]. Underreporting of the immunization status in pregnant women could also be contributing to the low coverage in our study (19% of women had unknown vaccination status). Influenza vaccination is recommended to women at any stage of pregnancy [4] and the offer and recommendation of the vaccine by health professionals is determinant to increase influenza vaccination among pregnant women[59, 60].

Seasonal influenza vaccination in Spain has proven to be effective in other risk groups, preventing severe cases and hospitalizations in the elderly [61], and the benefit of vaccination in this group has been well documented [62]. It is known that maternal influenza immunization prevents infection in pregnant women [51] and their infants [40], but evidence on the prevention of severe illness in this group is limited [30]. Some observational studies have suggested that influenza vaccination prevents preterm birth and foetal death [41], although more research is needed to assess the impact of maternal influenza immunization on birth outcomes [30, 39].

In this study, we aimed to estimate the potential effect of the seasonal vaccine in preventing influenza complications in pregnant women with confirmed influenza infection. The small sample size was a limiting factor for this analysis. The number of pregnant women hospitalized with severe influenza in Spain within the study period was 176, and of those pregnant, only five vaccinated cases were reported. Such extremely low influenza vaccine coverage did not allow us to obtain adjusted estimates by age, virus type/subtype and underlying medical conditions. The crude estimations in this study suggest a protective effect of the vaccine against influenza complications (ICU admissions or death), but these results are not adjusted for confounding factors and should be interpreted with caution, not allowing to make clear conclusions. Also, when comparing the characteristics of pregnant women by level of severity, vaccination uptake was lower in women with the most severe outcomes, although differences were not statistically significant.

A significant limitation of our study results from the selection of the study population. We obtained the information from the SHCIC surveillance system. All women included in the analysis not only were hospitalized due to the severity of their illness, but they also had to meet at least one of the criteria included in the severe case definition for surveillance (pneumonia, septic shock, acute respiratory distress syndrome, multiple organ dysfunction syndrome or ICU admission). As influenza severity was already high in all hospitalized women in the study, any potential protective effect of the vaccine against severe outcomes in pregnant women may have been underestimated in this already severely affected study population.

When compared to the non-pregnant group, we found that pregnant women were younger and healthier, which could have biased the results of the analysis of pregnancy as a risk factor for severe influenza outcomes (ICU and/or death). To control for this “healthy bias”, we adjusted the analysis for underlying medical conditions and age group, among other potential confounding factors.

In conclusion, we found that pregnant women with severe influenza in Spain have an increased risk of being hospitalized, but we found no evidence that pregnancy increases the risk of influenza severity in terms of ICU admission or death. Despite the mentioned limitations in the analysis, our results suggest that pregnant women could benefit from seasonal influenza vaccination, which has been shown to be effective in preventing severe complications in the elderly. Thus, our findings lend support to the WHO and Spanish official recommendations on influenza vaccination in pregnancy. In order to increase the quality of the SHCIC surveillance information for future studies with a larger sample size, improved reporting of pregnancy and vaccination status in all hospitalized confirmed influenza cases is recommended. Based on their increased hospitalization risk, pregnant women should continue to be considered a risk group for severe influenza. Additional efforts are required to increase vaccination uptake in all groups at risk of severe influenza in Spain, and particularly in pregnant women.

## Supporting information

S1 File Members of the Spanish Influenza Surveillance System (SISS). (DOCX)
