# Supplementary material for: Exploring the risk of severe outcomes and the role of seasonal influenza vaccination in pregnant women hospitalized with confirmed influenza, Spain, 2010/11-2015/16
Source: PLoS One. 2018 Aug 8;13(8):e0200934. doi: 10.1371/journal.pone.0200934 (PMC6082521; doi:10.1371/journal.pone.0200934)
Supplement: S1 File — (DOCX) [file pone.0200934.s001.docx]

**The Spanish Influenza Surveillance System (SISS) includes:**

Physicians of the influenza sentinel surveillance networks from Andalucía, Aragón, Asturias, Baleares, Canarias, Cantabria, Castilla-La Mancha, Castilla y León, Cataluña, Comunidad Valenciana, Extremadura, Madrid, Navarra, País Vasco, La Rioja, Ceuta and Melilla.

Andalucía: Virtudes Gallardo (Servicio de Epidemiología, Consejería de Salud); José María Navarro (Hospital Virgen de las Nieves de Granada). Aragón: Miriam García, Elisa Marco (Servicio de Vigilancia en Salud Pública, Dirección General de Salud Pública); Manuel Omeñaca (Hospital Universitario Miguel Servet). Asturias: Ismael Huertas (Dirección General de Salud Pública y Planificación, Consejería de Salud y Servicios Sanitarios); María de Oña (Hospital Universitario Central de Asturias de Oviedo). Baleares: Jaume Giménez (Servicio de Epidemiología, Dirección General de Salut Pública); Jordi Reina (Hospital Son Espases de Palma de Mallorca). Canarias: Eva Rivas Wagner, Lucas González (Servicio de Epidemiología y Prevención, Consejería de Sanidad); Carmen Pérez (Hospital Doctor Negrín de Las Palmas de Gran Canarias). Cantabria: Luis Viloria (Sección de Epidemiología, Consejería de Sanidad, Trabajo y Servicios Sociales); Mónica Gozalo (Hospital Universitario Marqués de Valdecilla). Castilla-La Mancha: Gonzalo Gutiérrez (Servicio de Epidemiología, Consejería de Sanidad). Castilla y León: Tomás Vega (Observatorio de Salud Pública, DGSP, Consejería de Sanidad); Socorro Fernández (Servicio de Epidemiología, DGSP, Consejería de Sanidad); Raúl Ortiz de Lejarazu (WHO- National Influenza Centre, Hospital Clínico Universitario de Valladolid). Cataluña: Nuria Torner (Subdirecció General de Vigilància i Resposta a Emergències en Salut Pública, Agència de Salut Pública, CIBERESP); María de los Ángeles Marcos (WHO- National Influenza Centre, Hospital Clínic de Barcelona). Comunitat Valenciana: Aurora López (Subdirección General de Epidemiologia y Vigilancia de la Salud, Conselleria de Sanitat); Francisco González (Subdirección General de Epidemiologia y Vigilancia de la Salud, y Sanidad Ambiental. DGSP, Conselleria de Sanitat Universal i Salut Publica); Concepción Gimeno (Consorci Hospital General Universitari de València). Extremadura: Julián Mauro Ramos (Subdirección de Epidemiología. Dirección General de Salud Pública, Servicio Extremeño de Salud); Guadalupe Rodríguez (Complejo Hospitalario San Pedro de Alcántara, Cáceres). Galicia: María Jesús Purriños (Dirección Xeral Saúde Pública de Galicia); Juan García Costa (Complejo Hospitalario Universitario de Orense); Sonia Pérez-Castro (Complejo Hospitalario Universitario de Vigo). Madrid: Luis García Comas (Servicio de Epidemiología. Dirección General de Salud Pública); Juan Carlos Galán (Hospital Universitario Ramón y Cajal). Murcia: Ana García Fulgueira (Servicio de Epidemiología, Consejería de Sanidad); Antonio Moreno (Hospital Virgen de la Arrixaca de Murcia). Navarra: Jesús Castilla (Sección de Vigilancia de Enfermedades Transmisibles del Instituto de Salud Pública, CIBERESP); Mirian Fernández-Alonso (Clínica Universidad de Navarra); Carmen Ezpeleta (Complejo Hospitalario de Navarra). País Vasco: Fernando González Carril (Servicio de Salud Pública, Departamento de Salud); Gustavo Cilla (Hospital Donostia de San Sebastián, CIBERES). La Rioja: Eva Martínez Ochoa, Carmen Quiñones (Servicio de Epidemiología y Prevención Sanitaria, Dirección General de Salud Pública y Consumo); Miriam Blasco (Hospital San Pedro de Logroño). Ceuta: Ana Rivas (Sección de Vigilancia Epidemiológica, Consejería de Sanidad y Bienestar Social); José López Barba (Hospital Universitario de INGESA). Melilla: Daniel Castrillejo (Servicio de Epidemiología, Dirección General de Sanidad y Consumo, Consejería de Bienestar Social y Sanidad). Francisco Pozo, Inmaculada Casas (National Centre for Microbiology, National Influenza Reference Laboratory, WHO-National Influenza Centre, ISCIII).
